# Physical Functioning, Perceived Disability, and Depressive Symptoms in Adults with Arthritis

**DOI:** 10.1155/2013/525761

**Published:** 2013-09-05

**Authors:** Katie Becofsky, Meghan Baruth, Sara Wilcox

**Affiliations:** ^1^Department of Exercise Science, University of South Carolina, Public Health Research Center, 921 Assembly Street, Columbia, SC 29208, USA; ^2^Saginaw Valley State University, University Center, College of Health and Human Services, MI 48710, USA; ^3^University of South Carolina, Prevention Research Center, Columbia, SC 29208, USA

## Abstract

This study investigated how physical functioning and perceived disability are related to depressive symptoms in adults with arthritis (*n* = 401). Participants self-reported depressive symptoms and disability. Objective measures of physical functioning included the 30-second chair stand test, 6-minute walk test, gait speed, balance, grip strength, and the seated reach test. Separate quantile regression models tested associations between each functional measure and depressive symptoms, controlling for age, gender, race, BMI, self-reported health status, and arthritis medication use. The association between perceived disability and depressive symptoms was also tested. Participants averaged 56.3 ± 10.7 years; 85.8% were women; 64.3% were white. Lower distance in the 6-minute walk test, fewer chair stands, slower gait speed, and greater perceived disability were associated with greater depressive symptoms in unadjusted models (*Ps* < 0.05). Fewer chair stands and greater perceived disability were associated with more depressive symptoms in adjusted models (*Ps* < 0.05). Balance, grip strength, and seated reach were not related to depressive symptoms. The perception of being disabled was more strongly associated with depressive symptoms than reduced physical functioning. To reduce the risk of depression in arthritic populations, it may be critical to not only address physical symptoms but also to emphasize coping skills and arthritis self-efficacy.

## 1. Introduction

For public health purposes, the term arthritis refers to over 100 musculoskeletal conditions of varying etiologies that cause pain, aching, or stiffness in or around a joint [1]. During 2007–2009, an estimated 50 million adults in the United States reported doctor-diagnosed arthritis [2]. As the US population grows in number and the baby boomers continue to enter older adulthood, arthritis is projected to affect 67 million Americans by 2030 [3]. When rising obesity rates are also considered, an even larger public health burden can be expected, as obesity has been associated with both the development and progression of arthritis [4].

A recent study estimates that 18% of adults with arthritis also have comorbid depression [5], compared with 7% of the general US population [6]. This high prevalence may be due, in part, to the functional limitations associated with arthritis symptoms. Depression may also be linked to perceived disability, a construct closely related to functional limitation, but with an important distinction: functional limitations alone, defined as alterations in the performance of a functional task, are not the equivalent of disability, defined as the inability of an individual to perform his or her social and environmental roles [7]. 

Previous research investigating the relationship between arthritis-attributable functional limitations and depression has largely assessed physical function with self-report measures. It is important to evaluate how individuals perceive their functional limitations, but it must be recognized that the presence of elevated depressive symptoms may influence how one reports their level of functioning and vice versa. This paper examined depressive symptoms in relation to both objective physical functioning and perceived disability in a diverse sample of adults with arthritis. We hypothesized that both would be significantly related to depressive symptoms. We also expected that perceived disability would be more strongly associated with depressive symptoms than any functional measure.

## 2. Methods

*STEPS to Health* was a randomized controlled trial with an attention control group. Participants were randomized to a 12-week self-directed multicomponent exercise program (First Step to Active Health) or to an attention control self-directed nutrition program (Steps to Healthy Eating). The trial was approved by the University of South Carolina's institutional review board. This paper used baseline data only (i.e., prior to randomization); therefore, a detailed description of the intervention is not described here. All analyses are cross-sectional.

Adults with arthritis were recruited from a four-county area in the midlands region of South Carolina with a relatively high percentage of ethnic minorities. The necessary sample size was estimated at *n* = 400 from power calculations to detect changes in the randomized trial. A number of recruitment strategies were used; worksite listservs and newspaper advertisements had the greatest yield. Interested participants contacted the study office and completed a telephone screening to assess eligibility status. 

Participants were eligible for *STEPS to Health* if they answered “yes” to the question: “Have you EVER been told by a doctor or other health care professional that you have some form of arthritis, rheumatoid arthritis, gout, lupus, or fibromyalgia?”. This question uses the Centers for Disease Control definition of arthritis and is used in a national surveillance survey [8]. Self-reporting of doctor-diagnosed arthritis has been validated for public health research purposes [1]. Participants were also eligible if they reported at least one symptom of arthritis (joint pain, stiffness, tenderness, decreased range of motion, redness and warmth, deformity, crackling or grating, and fatigue). Eligible participants additionally had no one else in their household participating in the study, were 18 years of age or older, were not planning to move out of the area in the next 9 months, were able to read and write in English, and were not participating in another research study (unless it was an observational study without an intervention or medication). 

Participants were ineligible for *STEPS to Health* if they had a fall in the past year that required medical assistance; were pregnant, breastfeeding, or planning to become pregnant in the next year (women); had diabetes and were taking insulin; could not walk longer than 3 minutes without taking a rest; could not stand without assistance for more than 2 minutes; could not sit in chair without arms for more than 5 minutes; or were already physically active (aerobic activities ≥3 days/week for ≥30 minutes/day or strength training ≥2 days/week for ≥20 minutes/day). The Physical Activity Readiness Questionnaire (PAR-Q) [9] was also administered, and participants were excluded if they were told by a health care provider that they had a heart condition and should only do exercise recommended by a doctor; experienced chest pain during rest or physical activity; experienced dizziness or loss of consciousness; had a bone or joint problem (besides arthritis) that could be made worse by physical activity; or knew of any other reason they should not do physical activity. Participants were not excluded if they took medication for hypertension; however, they were excluded if they had uncontrolled hypertension (≥160/100).

Participants who were deemed eligible following the telephone screening were scheduled to take part in a measurement session. A total of 24 baseline sessions with 6 to 30 participants in each were conducted from 3/27/2010 to 10/15/2011 to meet recruitment goals. Upon providing consent to participate, staff administered physical measurements including height, weight, and a number of physical function tests. Participants also brought in a survey that they had completed prior to the session. Participants received $20 cash incentive for taking part in the baseline measurement session.

### 2.1. Measures

#### 2.1.1. Sociodemographic/Health Related

Participants reported their age, gender, race, marital status, highest grade or years of education completed, and their overall health status. Height to the nearest quarter inch and weight to the nearest tenth kilogram were obtained. Body mass index (BMI) was calculated as kg/m^2^.

#### 2.1.2. Depressive Symptoms

The 10-item Center for Epidemiological Studies Depression Scale (CES-D) measured symptoms of depression [10]. On a scale of 0 (rarely or none of the time) to 3 (most or all of the time), participants rated the frequency with which they experienced 10 symptoms of depression during the past week. Responses were summed to yield a score ranging from 0 to 30, with a higher score indicating greater depressive symptoms. This measure has been shown to be reliable and valid [11].

#### 2.1.3. Medication

Participants reported if they were currently taking Tylenol or acetaminophen, nonsteroidal anti-inflammatory drugs (NSAIDs), COX-2 inhibitors, oral steroids, narcotic pain relievers, or any other over-the-counter and prescription medications for their arthritis (open-ended question). Medications listed in the open-ended questions were coded and reclassified to the above mentioned categories if applicable. Given the common reporting of the use of disease-modifying antirheumatic drugs (DMARDS) in the open-ended question, an additional category of drugs was created. If participants reported current or at least one day of use of one or more of these drugs in the past 7 days, they were considered to be using arthritis medication. 

#### 2.1.4. Upper Body Strength

Grip strength was measured (in kilograms) on the dominant hand using a Jamar hand dynamometer (Lafayette Instruments, Lafayette, IN). Participants stood with their dominant arm at their side (not touching the body), elbow bent to 90 degrees, wrist in the neutral position, and thumb superior. On the signal participants squeezed the dynamometer with as much force as possible. Participants were given one practice and three tests trials. The best of three trials was used as the score. This measure has been shown to be reliable and valid [12].

#### 2.1.5. Lower Body Strength

Lower body strength was measured using the 30-second chair stand test. Participants were directed to sit in the middle of a standard chair with their back straight, feet flat on the floor, and with their hands on the opposite shoulder crossed at the wrist. On the signal participants rose to a full stand and returned to a fully seated position, without using their arms. One practice of 1–3 repetitions was followed by one 30-second trial. The score is the total number of unassisted stands during the 30-second time frame. This measure has been shown to be valid and have good test-retest reliability in older adults [13].

#### 2.1.6. Lower Body Flexibility

Lower body flexibility was measured using the seated reach test. Participants removed their shoes and sat on a raised mat with their legs extended, knees straight, and feet positioned against the sit and reach box. With their arms outstretched, hands overlapping, and middle fingers even, participants slowly bent forward, reaching as far forward as possible toward their toes and pushing a marker forward. Participants were given two practices and three test trials. The score was the total distance reached to the nearest 0.5 cm, using the best of the three trials. This measure has shown acceptable validity (for hamstring flexibility) in middle-aged and older adults [14].

#### 2.1.7. Functional Exercise Capacity

The 6-minute walk test was used to measure functional exercise capacity. A 38-meter walking course was marked with cones in a level, carpeted hallway. Participants were instructed to walk as quickly as possible (not run) for six minutes. They were allowed to use their usual assistive devices during the test and were allowed to slow down, stop, or rest as necessary. The score was the distance walked (in meters) in six minutes. This test has been shown to be valid and reliable [15, 16].

#### 2.1.8. Gait Speed

The GAITRite (CIR Systems, Havertown, PA), a portable walking mat with software, was used to measure gait speed (in meters/second). Participants walked on the instrumented walkway without shoes at their normal walking pace. Participants completed three test trials, with the data averaged across the three trails. Participants needing an assistive device were allowed to use it during data collection. The GAITRite has been previously validated with a three-dimensional motion analysis system [17]. Test-retest reliability has also been shown to be high [18].

#### 2.1.9. Balance

Postural data was collected using an AMTI force platform. Participants stood quietly without shoes using a standardized foot position (closed stance) with their arms to the side. Participants completed one trial standing with eyes open and another standing with eyes closed. Each trial lasted 30 seconds. The total displacement of the center of pressure (COP_displacement_) was extracted to represent postural stability. A ratio of postural sway with eyes closed compared to eyes open was used as our performance indicator. Smaller ratios indicate greater postural stability or balance. In prior research, test-retest correlations for this measure were found to be high [19].

#### 2.1.10. Perceived Disability

The 20-item Health Assessment Questionnaire (HAQ) Disability Index was used to measure disability in eight categories of daily activities (i.e., dressing, arising, eating, walking, hygiene, reach, grip, and common activities). On a scale of 0 (without any difficulty) to 3 (unable to do), participants reported the amount of difficulty they had in performing two or three specific activities (in each category) over the past week. Each of the eight categories was assigned a score based on the highest score of any activity within the category. If the category score was lower than a 2, but a participant reported usually using a device or aid to perform the activity, the score was increased to a 2. The total score was the mean of the eight categories. Scores ranged from 0 to 3, with a higher score indicating higher impairment. This measure has been shown to be valid and reliable [20].

### 2.2. Statistical Analyses

Basic descriptive statistics included frequencies and means of key survey, selected demographic, and health-related variables. Traditional linear regression models (PROC GLM) violated normality assumptions due to a high percentage of participants reporting low values for depressive symptoms. Thus, quantile regression models (PROC QUANTREG), which model the median rather than the mean, examined the relationship between physical functioning and self-reported disability (independent variables) with depressive symptoms (dependent variable). We conducted a separate model for each physical function measure (i.e., 30-second chair stand, 6-minute walk test, seated reach, grip strength, gait speed, and balance) and self-reported disability. Covariates were age, gender, race (White or non-White), BMI, self-reported health status, and medication use (yes/no). All *P* values were 2-sided with an alpha level of 0.05. We performed all data analyses using SAS version 9.2 (SAS Institute, Inc., Cary, North Carolina). 

## 3. Results

The sample was comprised of 401 adults with arthritis. Participant characteristics are presented in Table 1. Most participants were women (85.8%), 64.3% were white, and 35.5% were African American. Approximately half of the sample reported having osteoarthritis (50.4%), with rheumatoid arthritis (21.7%) and fibromyalgia (18.2%) the next most common types of arthritis.  Table 2 includes mean scores for the independent variables of interest and median and range scores for depressive symptoms. 

Table 3 shows the results of the unadjusted and adjusted quantile regression models. In the unadjusted models, lower distance walked in the 6-minute walk test (*P* = 0.004), fewer chair stands (*P* < 0.001), slower gait speed (*P* = 0.004), and greater perceived disability were associated with greater depressive symptoms. There was no relationship between depressive symptoms and grip strength, seated reach, and balance (*Ps* ≥ 0.05). In the adjusted models, fewer chair stands (*P* = 0.02) and greater perceived disability (*P* < 0.001) were associated with more depressive symptoms. Six-minute walk performance, gait speed, grip strength, seated reach, and balance performance were all unrelated to depressive symptoms (*Ps* ≥ 0.05). 

## 4. Discussion 

Of the nearly 50 million people reporting arthritis in 2007–2009, almost half (21 million) reported arthritis-attributable activity limitation [2]. Previous studies have shown that individuals with functional limitations are at an increased risk for depression [21], which is associated with an increased risk of cardiovascular disease [22] and greater risk of mortality in multiple medical conditions [23, 24]. The purpose of this study was to investigate the relationship of physical functioning and perceived disability with depressive symptoms in adults with arthritis. This paper strengthens and extends the current knowledge base due to its diverse sample and use of objective measures of physical functioning.

A number of physical performance measures (i.e., 6-minute walk performance, gait speed, and chair stands) and perceived disability were associated with depressive symptoms in the unadjusted models. However, 6-minute walk performance and gait speed were no longer significant in the adjusted models. Many of the covariates (e.g., age, BMI, and self-rated health) were highly correlated with many of the physical performance measures, as well as with depressive symptoms. Therefore, true relationships between physical functioning and depressive symptoms may have been masked. The significant relationship between chair stands and depressive symptoms in the adjusted model suggests that this measure may be a particularly strong indicator of mental health status, perhaps because lower body strength is most essential to completing activities of daily living and maintaining independence. A previous study also showed an association between depression and performance on the 30-second chair stand task in adults with fibromyalgia [25]. 

The main finding from this study was that perceived disability was more strongly associated with depressive symptoms than objective physical performance. Our findings reflect the conclusions drawn from a recent study of older adults with mobility limitations: performance-based and patient-report measures of function do not provide equivalent information, as self-report measures assess a much broader range of factors [26]. Indeed, it seems intuitive that a perception that one's daily activities are being compromised may be more directly linked with depression than a physical limitation that may or may not interfere with valued activities. 

 It should be emphasized that the relationship between physical functioning and depression is a complex, often self-perpetuating cycle. Depression caused by arthritis-attributable activity limitation may contribute to a more rapid decline of physical health, possibly by decreasing physical activity levels and reducing adherence to medication [27]. As physical health worsens, mental health may continue to deteriorate. A similar bidirectional relationship exists between arthritis-related pain and depression [27]. Ormel and colleagues [28] used structural equation modeling to examine the temporality of the disability-depression relationship, finding that poor physical health (including illness and disability) quickly and strongly affected depressive symptoms, whereas depressive symptoms had a delayed and weaker effect on physical health. 

 The primary limitation of this study is its cross-sectional design, limiting causal inferences. Although arthritis is more prevalent in women than men, our study is also limited by the small percentage of male participants (14.2%). Participants were also relatively high functioning with low levels of depressive symptoms, which suggests that associations between physical functioning and depressive symptoms may be stronger than reported in this paper. Finally, we do not know what percentage of our sample was taking antidepressant medication; unfortunately this information was not collected. Strengths of this study include the large, racially diverse sample and use of both subjective and objective measures. 

 Our findings have implications for the design of future studies, suggesting that objective and subjective measures are complementary rather than redundant, and ideally both should be used. Additionally, prospective studies that follow individuals with arthritis without comorbid depression at baseline (or that follow depressed individuals without physical limitations at baseline) would help in verifying the primary directionality of the relationship. Once directionality is established, it may be useful to conduct mediation analyses to determine what mechanism most strongly links functional limitation and disability with depression (e.g., low physical activity levels, low self-efficacy) in order to inform interventions. Treating depression in individuals with comorbid depression and osteoarthritis has been shown to improve physical functioning, decrease pain severity, and increase overall quality of life at 12 months [29]. Physical activity interventions appear very promising, as physical activity has the potential to not only soothe symptoms of arthritis but also improve physical function and mental health [30]. Six physical activity programs have already been proven safe and effective for adults with arthritis [1]. Interventions should also determine whether fostering self-efficacy and self-management skills reduces depressive symptoms [31]. 

## 5. Conclusions

 Overall, this baseline analysis suggests that the perception of being disabled may be more strongly associated with depressive symptoms than reduced physical functioning alone in adults with arthritis. It seems logical that a conscious awareness that one's condition is interfering with daily activities would be more telling of mental health status than a simple evaluation of physical performance. To reduce the risk of depression in adults with arthritis, it may be critical not only to alleviate the physical symptoms contributing to functional limitation but also to teach coping skills that can help physically limited individuals maintain their daily routine as much as possible. 

## Figures and Tables

**Table 1:** Demographic and health-related characteristics of participants (*n* = 401).

	*N *	Mean (SD), range, or %
Age, years	401	56.3 (10.7), 19–87
Gender		
Women	344	85.8
Men	57	14.2
Race		
White	256	64.0
Black/African American	141	35.3
Other race	3	0.8
Education level		
<High school	6	1.5
High school or GED	46	11.5
Some college	105	26.3
College graduate	243	60.8
Marital status		
Married or member of unmarried couple	243	60.6
Divorced or separated	71	18.0
Widowed	36	9.0
Never married	50	12.5
Body mass index, kg/m^2^	401	33.0 (8.2), 15.8–60.7
Weight group		
Underweight	1	0.3
Normal weight	58	14.5
Overweight	114	28.4
Obese	228	56.9
Self-reported arthritis type^a^		
Osteoarthritis	202	50.4
Rheumatoid arthritis	87	21.7
Fibromyalgia	73	18.2
Gout	31	7.7
Lupus	13	3.2
Sjogren's disease	9	2.2
Psoriatic arthritis	8	2.0
Other	11	2.8
Don't know	65	16.3
Arthritis medication usage (current or past 7 days)		
Yes	341	85.0
No	60	15.0
Self-reported health status		
Excellent	12	3.0
Very good	102	25.5
Good	199	49.8
Fair	74	18.5
Poor	13	3.3

^a^Percentages for self-reported arthritis exceed 100% as participants could report >1 type of arthritis.

**Table 2:** Descriptive statistics for independent and dependent study variables.

Independent variables	*N *	Mean (SD)
Grip strength (kg)	401	27.1 (10.2)
6-minute walk distance (m)	399	494.1 (91.2)
30-second chair stands (#)	401	10.0 (3.5)
Seated reach (cm)	399	21.7 (9.9)
Gait velocity (m/sec)	399	1.1 (0.2)
Balance^a^ (ratio of eyes open/eyes closed)	398	1.2 (0.3)
HAQ score (0–3)	401	0.6 (0.5)

Dependent variable	*N *	Median, Range

Depressive symptoms^a^ (0–30)	401	5, 0–28

HAQ: Health Assessment Questionnaire Disability Index.

^a^Lower score = more favorable.

**Table 3:** Relationships between physical functioning and depressive symptoms.

	Model 1^a^			Model 2^b^		
	Intercept (SE)	Estimate (SE)	*P* value	Intercept (SE)	Estimate (SE)	*P* value
Grip strength	5.00 (1.20)	0.00 (0.04)	1.00	3.27 (2.14)	−0.05 (0.03)	.05
6-minute walk	10.12 (1.80)	−0.01 (0.00)	.004	5.26 (4.02)	−0.01 (0.00)	.16
Chair stands	8.10 (0.84)	−0.30 (0.08)	<.001	5.22 (2.26)	−0.22 (0.09)	.02
Seated reach	5.00 (0.69)	0.00 (0.03)	1.00	1.98 (1.96)	−0.02 (0.03)	.41
Gait speed	8.81 (1.28)	−3.34 (1.15)	.004	4.65 (2.93)	−1.86 (1.22)	.13
Balance	3.35 (2.00)	1.50 (1.72)	.39	.57 (1.78)	1.05 (1.37)	.45
HAQ	3.33 (0.44)	3.33 (0.60)	<.001	3.77 (1.89)	2.41 (0.59)	<.001

HAQ: Health Assessment Questionnaire Disability Index.

^a^Model 1 unadjusted model.

^b^Model 2 adjusted model; controlled for gender, age, race, body mass index, self-rated health, and arthritis medication use.
